# Neonatal Renal Failure in the Setting of Anorectal Malformation: A Case Report and Literature Review

**DOI:** 10.7759/cureus.14984

**Published:** 2021-05-12

**Authors:** Wendy Jo Svetanoff, Asma Ahmed, Richard J Hendrickson, Rebecca M Rentea

**Affiliations:** 1 Pediatric Surgery, Children's Mercy Hospital, Kansas City, USA; 2 General Surgery, University of Missouri Kansas City School of Medicine, Kansas City, USA; 3 Pediatric Surgery, University of Missouri Kansas City School of Medicine, Kansas City, USA

**Keywords:** anorectal malformation, end stage renal disease, pediatric surgery, colorectal, urinary anomaly, vacterl, lower gi surgery, colorectal surgery

## Abstract

Anorectal malformations (ARMs) can occur in isolation or in association with other anomalies, most commonly those of the genitourinary systems. Morbidity and mortality are highest among patients who develop end-stage renal disease (ESRD) either from severe congenital anomalies (dysplastic kidneys) or from repeated infections in those who have vesicoureteral reflux or persistent recto-urinary fistulas. We describe our management strategy for a patient born with an ARM and bilateral dysplastic kidneys to highlight the nuances and complex decision-making considerations required in taking care of this complex patient population.

Our patient is a male twin born at 32 weeks’ gestational age who was found to have bilateral dysplastic kidneys on prenatal ultrasound. On initial examination, an imperforate anus was identified along with a severe urethral stricture. Full workup also revealed sacral dysgenesis and confirmation of the dysplastic kidneys. On day of life 3, a laparoscopic diverting sigmoid colostomy was performed; urologic evaluation confirmed the severe urethral stricture, which required dilation to place an 8F council tip catheter. Due to his small size, peritoneal dialysis could not be initiated until five weeks of age. As full volumes could not be reached with peritoneal dialysis, he was soon transitioned to continuous renal replacement therapy.

At five months of age, a laparoscopic-assisted posterior sagittal anorectoplasty (PSARP) was performed. As his urethral stricture had worsened, a suprapubic catheter had been placed for bladder decompression. Reversal of his colostomy was performed 15 days after PSARP. Unfortunately, the patient required three further surgical interventions due to abdominal wall and inguinal hernias contributing to filling and emptying dysfunction when utilizing peritoneal dialysis. He is currently 16 months of age and remains inpatient due to intermittent hemodialysis requirements along with autocycling of his peritoneal dialysis. He is working on developmental milestones, can pull to a stand, and is currently being evaluated for kidney transplantation.

The development of ESRD in a neonate or infant with an ARM is rare and can be due to congenital dysplasia or agenesis of bilateral kidneys. While peritoneal dialysis is the preferred approach, catheter dysfunction can result from intra-abdominal adhesions or inadequate fluid removal from inguinal or abdominal wall hernias that form in the setting of increased intra-abdominal pressure required for peritoneal dialysis. Close collaboration is required between pediatric surgeons, nephrologists, and urologists to facilitate colonic and urologic reconstruction and manage catheter-related complications.

## Introduction

Anorectal malformations (ARMs) are developmental anomalies involving the distal anus and rectum. An ARM can occur in isolation or in association with other anomalies as part of the VACTERL association. ARMs include a wide spectrum of anomalies ranging from a simple perineal fistula or anal stenosis to a complex cloacal anomaly where the colonic, urinary, and gynecologic systems fail to separate [1]. The presence of genital or urologic anomalies ranges from 25% to 60% in the ARM population and increases in incidence with the complexity of the ARM [1,2]. Patients with concomitant ARMs and urological anomalies have the highest morbidity and mortality due to the development of end-stage renal disease (ESRD) either from the renal anomaly itself (i.e., dysplastic kidneys) or from repeated infections such as vesicoureteral reflux or persistent recto-urinary fistula remain [1]. The mortality rate of patients with ARM who develop ESRD ranges from 2.5% to 6%, warranting the need for early and innovative interventions [3].

A posterior sagittal anorectoplasty (PSARP) is the primary reconstructive option for patients with ARM; this can be performed as a primary single-stage procedure or as a staged operation with the use of a diverting colostomy as the initial step [4]. In small children with ESRD, peritoneal dialysis (PD) is the preferred choice as it facilitates nutrition and fluid management and is often better tolerated than hemodialysis (HD). However, PD catheter placement in a peritoneum that is already breached by a colostomy enhances the probability of complications, including catheter-associated infections [3,5]. Thoughtful, expeditious, and complex measures are needed to avoid and manage complications in these newborns. In this case report, we describe our management strategy for a newborn male with ARM and dysplastic kidney, leading to early ESRD and the need for PD.

## Case presentation

**Figure 1:**
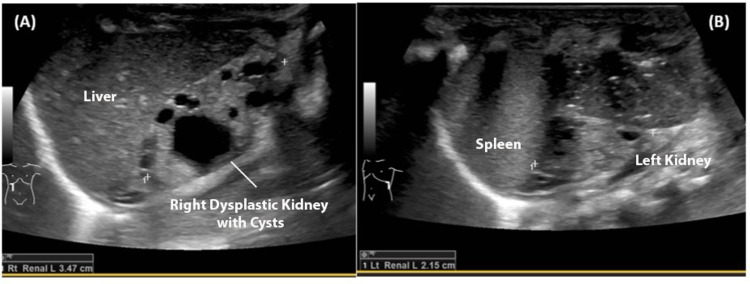
Ultrasound images of the right (A) and left (B) dysplastic kidneys.

The patient was a preterm male twin who was born at 32 weeks’ gestational age via cesarean section due to non-reassuring fetal heart tones. Birth weight was 1.6 kg and initial Apgar scores were 4 and 8 at 1 minute and 5 minutes, respectively. A prenatal renal ultrasound revealed bilateral dysplastic kidneys. Due to respiratory distress, the infant was intubated in the delivery room and given surfactant. On initial examination, an imperforate anus was noted with no signs of a perineal fistula. An echocardiogram showed a structurally normal heart; however, spinal radiographs revealed sacral dysgenesis and hemivertebrae with a fusion of S4 and S5. Repeat renal imaging confirmed the presence of nonfunctioning bilateral dysplastic kidneys without hydroureteronephrosis to suggest obstruction (Figures 1A, 1B). A Foley catheter was unable to be passed by the urology team due to a severe urethral stricture.

On day of life 3, the patient was taken to the operating room, where a laparoscopic diverting sigmoid colostomy with mucus fistula was performed. Once again, a Foley catheter was unable to be passed through the urethra at the time of the operation. Thus, after the colostomy was formed, antegrade cystoscopy was performed through the laparoscopic umbilical incision. Findings included significant urethral narrowing, which required dilation before an 8F council tip Foley catheter placement. No signs of a urethral-rectal fistula were identified.

Due to his small size and unknown urinary functional status, he was deemed not to be a candidate for PD catheter placement at the time of the initial operation. Over the following weeks, his renal function was managed with strict fluid goals and sodium chloride and bicarbonate supplementation. Upon return of bowel function, the patient was started on breast milk feeds, which was later alternated with Renastart™ formula due to increasing potassium levels. The patient also required three albumin transfusions due to increasing periorbital and lower extremity edema.

**Figure 2:**
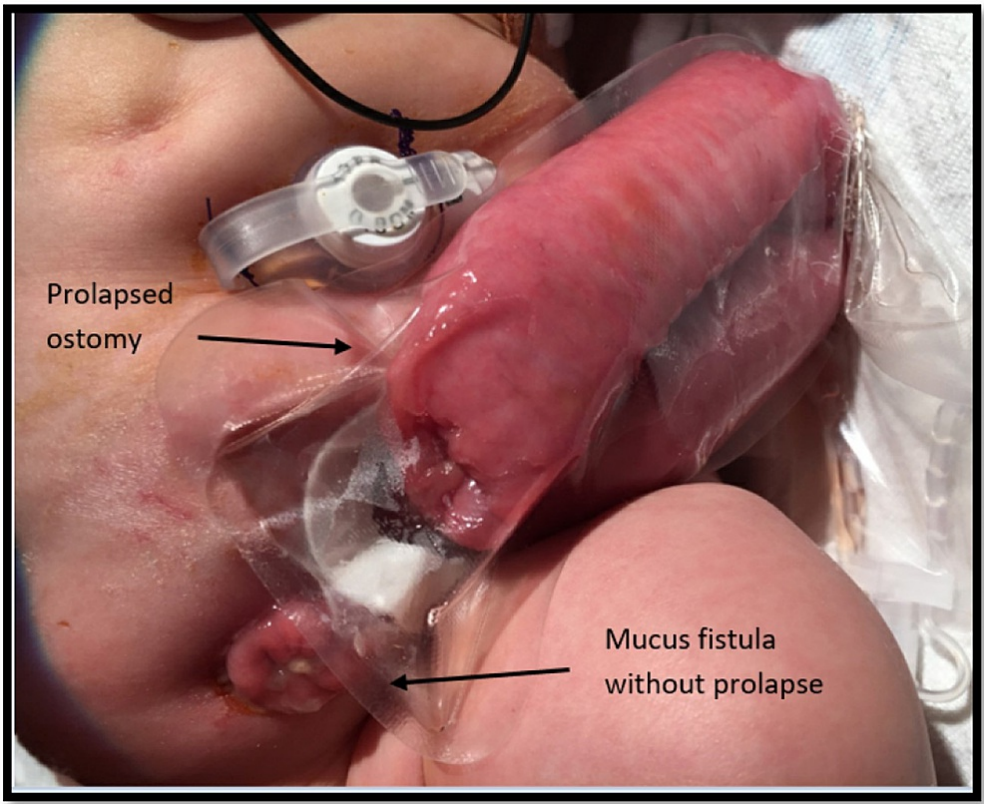
Prolapse of sigmoid colostomy. The patient developed prolapse of his end sigmoid colostomy. Although previous attempts successfully reduced the prolapse at bedside, this prolapse was reduced in the operating room and colopexy sutures were placed to prevent further occurrences. This was done in conjunction with a gastrostomy tube placement.

At five weeks of age, he underwent PD placement in anticipation of requiring dialysis due to worsening renal function. He successfully began PD but could not reach full volumes, requiring a transition to HD with continuous renal replacement therapy (CRRT). He also developed prolapse of both his colostomy and mucus fistula, requiring reduction at the bedside and necessitating the placement of colopexy sutures (Figure 2). After removing the intra-operatively placed Foley catheter at five weeks postoperatively, the patient was unable to drain his bladder. Complete urethral atresia was noted, and a suprapubic catheter was placed.

**Figure 3:**
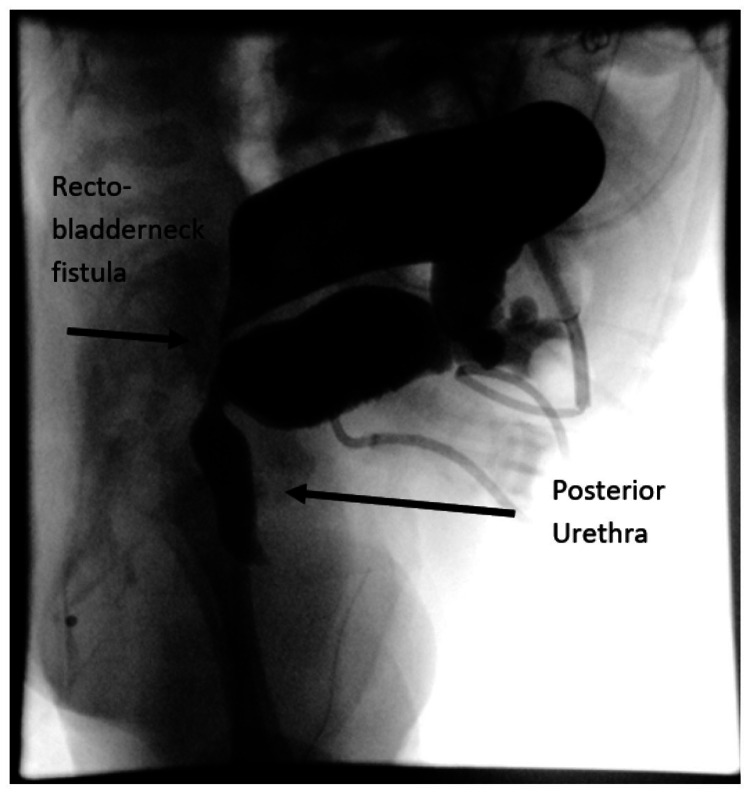
High-pressure distal colostogram revealing a recto-bladder neck fistula.

A pre-operative contrast evaluation revealed a high recto-bladder neck fistula (Figure 3). Thus, at five months of age, he underwent a laparoscopic-assisted PSARP, repair of left inguinal hernia, and exchange of the nonfunctioning suprapubic tube.

Through laparoscopy, the inguinal hernia was repaired, a fibrin plug was removed from the existing PD catheter, the rectum was mobilized, and the recto-bladder neck fistula was isolated and ligated. The completion of the anorectoplasty was performed via a posterior sagittal approach. An 11/12 size Hegar dilator was able to be placed in the neoanus without difficulty after the procedure. The colostomy was left in place; however, reversal was performed 15 days after PSARP to facilitate return to PD.

**Figure 4:**
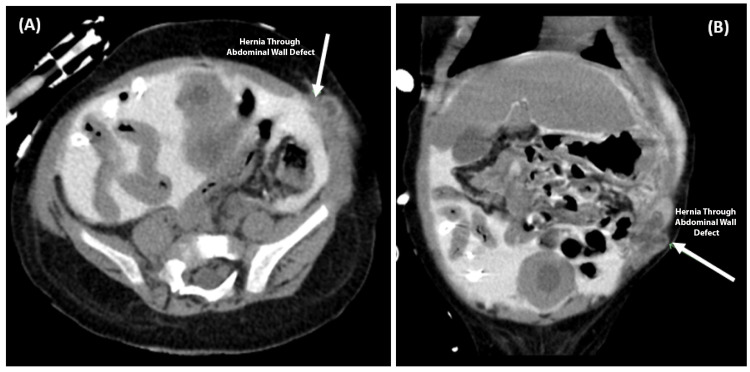
CT scan revealing an abdominal wall hernia at the site of a previous colostomy. Images are in the axial (A) and coronal (B) planes. Contrast was injected through the peritoneal dialysis catheter to determine areas of fluid accumulation that could explain the filling and emptying dysfunction noted during dialysis. The white arrows signify the abdominal wall hernia.

Since his ARM repair, the patient, unfortunately, has had continued problems with PD due to filling/emptying dysfunction, requiring HD to supplement the PD. At six months of age (one month after PSARP), he underwent PD catheter revision, where the catheter was found to be encased in pelvic adhesions, along with recurrent left inguinal hernia repair. At 10 months of age, he underwent laparoscopic repair of an abdominal wall hernia found at the previous colostomy site, an abdominal wall hernia found at a previous laparoscopic trocar site, umbilical hernia repair, and PD catheter revision (Figures 4A, 4B).

At 12 months of age, he underwent repair of a swiss-cheese hernia defect noted both at the previous colostomy site and near the gastrostomy tube area. No inguinal hernias were noted at this final intervention, and the PD catheter did not require revision.

**Figure 5:**
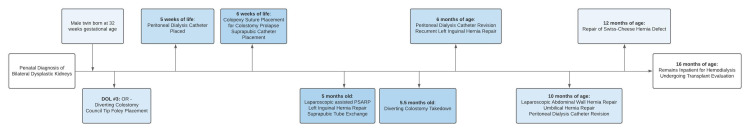
Timeline of events. This timeline shows the different surgical procedures that occurred throughout the patient's hospital stay following birth. DOL, day of life

At 16 months of age, the patient remains inpatient but has been transferred from the intensive care unit to the inpatient dialysis floor. At this point, his PD catheter is functioning well, and autocycling of his PD has begun; however, he still requires intermittent HD twice weekly. He is working on his developmental milestones, enjoys playing with staff and providers, and can pull to a stand. He has been tolerating gastrostomy feeds without difficulty and is working on oral skills with pureed feeds. His hospital course had also been complicated by multiple infections, including *Serratia* and *Enterococcus* urinary tract infections and urosepsis and enterococcus peritonitis, all of which have resolved; he remains on cephalexin for renal prophylaxis. He developed a nonocclusive thrombus of his inferior vena cava and agammaglobulinemia, for which he required intravenous immunoglobulin. He has been discharged home but remains on levothyroxine for hypothyroidism and growth hormone replacement. An initial evaluation for kidney transplantation has been completed, with plans to undergoing a living donor transplant in the future (Figure 5).

## Discussion

In this case report, we highlighted the rare occurrence of a patient with bilateral dysplastic kidneys, leading to early ESRD, in the setting of an associated ARM, and the management challenges of maintaining PD in an abdomen that has had multiple surgical interventions. The timing of PD and/or HD concerning ARM reconstruction is not well-defined. It can be fraught with complications, including the need for PD catheter revision or difficulties obtaining adequate fluid removal, both of which our patient encountered. In our patient, subsequent recurrent operative procedures were required to repair incisional and inguinal hernias, as these areas collected fluid during dialysis, preventing drainage and thus requiring supplemental HD.

Initial management of ARM

ARMs incorporate a wide range of presentations, ranging from low-lying anomalies, such as a perineal fistula, that can undergo reconstruction soon after birth, to high anomalies, such as a recto-bladder neck fistula or cloaca, in which a colostomy is initially performed for fecal diversion from the urinary system and full reconstruction occurs at a later date [3]. Factors that influence the timing and the success of the ARM repair include the infant's size at reconstruction, neurological status, the presence of associated anomalies, the experience of the surgeon, and the development of postoperative complications [4].

Colostomy and colostomy prolapse management

The creation of a colostomy before definitive reconstruction of an ARM ensures fecal diversion in those with a high level of the recto-urinary fistula, to protect the urinary system, to allow for patient growth before repair, and to allow time to complete a full anatomic workup. The full anatomic workup and imaging include a contrast study through the mucus fistula to adequately identify the recto-urinary fistula level [6]. The placement of a colostomy can be done through an open or laparoscopic approach. Multiple techniques for diversion are available, including an end colostomy for high ARMs, a divided sigmoid colostomy with mucus fistula (which allows for a fistulogram contrast study to identify the level of the rectourinary fistula before reanastomosis), and a loop colostomy (typically performed at the junction of the descending and sigmoid colon and can also be used to identify the rectourinary fistula later) [7]. However, nearly 40% of patients with colostomies present with complications, regardless of the type of colostomy used, including peristomal excoriations, prolapse, parastomal hernias, leakages, intra-abdominal adhesions, and bowel obstruction [4,7]. The patient in our case suffered a colostomy prolapse due to the increased intra-abdominal pressure from PD, which required multiple reductions, including the placement of a colostomy cerclage suture. Untreated prolapse can lead to ischemia of the prolapsed segment requiring bowel resection, and this loss in bowel length is unfavorable for further reconstructive surgeries for ARM. Nonsurgical management of colostomy prolapse has high recurrence rates, especially in cases where intra-abdominal pressure increases cannot be avoided [8]. Thus, many surgical techniques, such as stoma revision, transabdominal suture colopexy, and colostomy cerclage, have been advocated to treat colostomy prolapse [6,8]. Laparoscopic transabdominal colopexy has demonstrated great success rates in neonates. It does not require peritoneal dissection and is associated with minimal adhesion formation, thus preserving the option for laparoscopy during ARM reconstruction. Additionally, the thin abdominal wall of neonates favors quick and safe transabdominal fixation, minimizing operative time [6,8].

Associated urological anomalies

Urological anomalies, particularly hydronephrosis due to vesicoureteral reflux, are commonly associated with ARM [2]. Early identification and management are important to prevent or delay progression to ESRD. The initial diagnostic modality is a renal ultrasound, with a VCUG (voiding cystourethrogram) often used to assess reflux severity [1]. To further evaluate renal function, renal scintigraphy can be performed [9]. In patients with urethral stricture, urethral dilatation is the primary management mode; however, the success rate is only 20-30%. Often, a suprapubic catheter is required in patients. Placement of a suprapubic cystostomy allows for bladder decompression; intermittent closure of the tube allows for the assessment of bladder emptying while maintaining access to the urinary system [10]. Following urinary diversion, definitive treatment options for urethral strictures include wire-guided dilation, direct vision internal urethrotomy, urethroplasty, and open surgery [10]. Appropriate bladder emptying should be ensured with the use of a vesicostomy or clean intermittent catheterizations, and surgical correction of all urological abnormalities is necessary before undergoing renal transplant [10,11].

ARM and ESRD

The main cause of mortality in patients with ARM is more often due to complications from associated malformations than from the ARM itself. While cardiovascular malformations are the underlying cause of death at an early age, genitourinary malformations (found in 40-50% of the patients) lead to extensive morbidity and mortality in the later stages of life [1,2]. Genitourinary malformations can result in kidney injury, either from congenital dysplasia or from current or unrecognized vesicoureteral reflux and hydronephrosis [3]. This results in progressive damage that leads to ESRD, requiring dialysis. Up to 2% of patients with ARM and ESRD will ultimately require renal transplantation, with those who have a high ARM or cloaca being most at risk [3].

PD is the most common and preferred modality of dialysis for children younger than two years of age. The peritoneum not only has the advantage of being a natural biocompatible membrane for dialysis but also has a larger surface area in children than compared to adults, allowing a more efficient solute clearance [11]. However, there are incidences (size of the child, recent intra-abdominal surgery) where PD is contra-indicated and HD is required. HD is not the preferred option in children due to technical problems in obtaining and maintaining vascular access, development of vascular thrombi at the site of catheter insertion or elsewhere in the body, need for anticoagulation, and hemodynamic instability observed in neonates or infants who require HD [1]. Thus, for patients who require HD in the first few months of life, like our patient, CRRT is necessary due to the large fluid shifts and hemodynamic swings associated with intermittent HD, which requires admission to the intensive care unit for continuous monitoring and care. Even as an outpatient, intermittent HD requires travel to a dialysis center or hospital multiple times a week, which places a large burden on the patient's family compared with PD, which can be performed at home [1].

ARM and colostomy management with PD

The management of ARM with concomitant ESRD can be challenging and requires a multidisciplinary collaboration with pediatric surgeons, urologists, nephrologists, and neonatal/pediatric intensive care teams. Colostomies may be performed prior to or at the time of PD catheter insertion. During PD catheter placement, total omentectomy is performed to prevent catheter obstruction and malfunction. In infants with ARM, their small body size makes it conducive for a single-cuff straight catheter with a straight tunnel to be placed with an exit site situated as far away as possible from the colostomy site in the pre-sternal area or as high as possible in the right upper quadrant, contralateral to the colostomy site. Performing PD catheter placement and colostomy formation in the same surgery is beneficial as the common left lower abdominal incision for the placement of a colostomy can also be used to perform omentectomy as well as PD catheter placement under direct vision and in the right position. Multiple-layer closure is used for the wounds, and, if possible, it is recommended to wait a few days before the first session of dialysis [5].

However, although PD is the most preferred treatment for ESRD in infants, the presence of a colostomy increases the probability of developing PD-related complications [5]. Conventionally, PD was not initiated in patients with a colostomy due to the increased risk of peritonitis and catheter contamination. An attempt to increase the dwell time to the desired volume during PD can lead to increased intra-abdominal pressure resulting in colostomy prolapse and/or the development of umbilical hernias, inguinal hernias, or abdominal hernias at the site of previously healed incisions, all of which were encountered in our patient [11]. These complications lead to poor drainage of the dialysis fluid, which impacts the overall efficiency of dialysis. The International Pediatric Peritoneal Dialysis Network's current recommendation is for the PD catheter to exit on the side opposite from the colostomy site [5]. This can be optimized with laparoscopic placement, allowing visual guidance in directing the catheter away from any already-formed adhesions. According to Chadha et al., the laparoscopic placement of the dialysis catheter in the pre-sternal position is the most preferred site due to the distance away from the colostomy site and the decreased number of catheter-related infections compared to other sites for placement [12].

## Conclusions

The development of ESRD in a patient with an ARM is rare during the neonatal or infant period as this would only occur in the setting of congenital dysplasia or agenesis of bilateral kidneys. While PD is the preferred approach, this can be complicated by catheter dysfunction from intra-abdominal adhesions or inadequate fluid removal due to hernia formation in the setting of increased intra-abdominal pressure required for full-volume PD. A close collaboration between pediatric surgeons, urologists, and nephrologists is required to facilitate alternating use of PD and HD around colonic and urologic reconstruction and the management of catheter-related complications.
